# Brachial Artery Responses to Ambient Pollution, Temperature, and Humidity in People with Type 2 Diabetes: A Repeated-Measures Study

**DOI:** 10.1289/ehp.1206136

**Published:** 2014-01-07

**Authors:** Antonella Zanobetti, Heike Luttmann-Gibson, Edward S. Horton, Allison Cohen, Brent A. Coull, Barbara Hoffmann, Joel D. Schwartz, Murray A. Mittleman, Yongsheng Li, Peter H. Stone, Celine de Souza, Brooke Lamparello, Petros Koutrakis, Diane R. Gold

**Affiliations:** 1 Department of Environmental Health, Harvard School of Public Health, Boston, Massachusetts, USA; 2 Joslin Diabetes Center, Boston, Massachusetts, USA; 3 Department of Biostatistics, Harvard School of Public Health, Boston, Massachusetts, USA; 4 IUF Leibniz Research Institute for Environmental Medicine and Medical School, University of Düsseldorf, Düsseldorf, Germany; 5 Beth Israel Deaconess Medical Center, Harvard Medical School, Boston, Massachusetts, USA; 6 Department of Medicine, Brigham and Women’s Hospital, Harvard Medical School, Boston, Massachusetts, USA; 7 Channing Laboratory, Department of Medicine, Brigham and Women’s Hospital, Harvard Medical School, Boston, Massachusetts, USA

## Abstract

Background: Extreme weather and air pollution are associated with increased cardiovascular risk in people with diabetes.

Objectives: In a population with diabetes, we conducted a novel assessment of vascular brachial artery responses both to ambient pollution and to weather (temperature and water vapor pressure, a measure of humidity).

Methods: Sixty-four 49- to 85-year-old Boston residents with type 2 diabetes completed up to five study visits (279 repeated measures). Brachial artery diameter (BAD) was measured by ultrasound before and after brachial artery occlusion [i.e., flow-mediated dilation (FMD)] and before and after nitroglycerin-mediated dilation (NMD). Ambient concentrations of fine particulate mass (PM_2.5_), black carbon (BC), organic carbon (OC), elemental carbon, particle number, and sulfate were measured at our monitoring site; ambient concentrations of carbon monoxide, nitrogen dioxide, and ozone were obtained from state monitors. Particle exposure in the home and during each trip to the clinic (home/trip exposure) was measured continuously and as a 5-day integrated sample. We used linear models with fixed effects for participants, adjusting for date, season, temperature, and water vapor pressure on the day of each visit, to estimate associations between our outcomes and interquartile range increases in exposure.

Results: Baseline BAD was negatively associated with particle pollution, including home/trip–integrated BC (–0.02 mm; 95% CI: –0.04, –0.003, for a 0.28 μg/m^3^ increase in BC), OC (–0.08 mm; 95% CI: –0.14, –0.03, for a 1.61 μg/m^3^ increase) as well as PM_2.5_, 5-day average ambient PM_2.5_, and BC. BAD was positively associated with ambient temperature and water vapor pressure. However, exposures were not consistently associated with FMD or NMD.

Conclusion: Brachial artery diameter, a predictor of cardiovascular risk, decreased in association with particle pollution and increased in association with ambient temperature in our study population of adults with type 2 diabetes.

Citation: Zanobetti A, Luttmann-Gibson H, Horton ES, Cohen A, Coull BA, Hoffmann B, Schwartz JD, Mittleman MA, Li Y, Stone PH, de Souza C, Lamparello B, Koutrakis P, Gold DR. 2014. Brachial artery responses to ambient pollution, temperature, and humidity in people with type 2 diabetes: a repeated-measures study. Environ Health Perspect 122:242–248; http://dx.doi.org/10.1289/ehp.1206136

## Introduction

Previous studies have reported that associations of cardiovascular mortality with particle pollution (Zanobetti and Schwartz 2001; Zanobetti et al. 2009) and extreme ambient temperatures during heat waves (Medina-Ramón et al. 2006; Schifano et al. 2009; Schwartz 2005) are stronger in persons with diabetes than in those without diabetes. It has been hypothesized that people with diabetes are at greater risk for acute environmental perturbations of vascular (including coronary artery) function because of chronic endothelial dysfunction, autonomic dysregulation, atherosclerosis, or dysregulation of fluid balance and the renin–angiotensin system (Pop-Busui 2010; Ribeiro-Oliveira et al. 2008). Common medications such as angiotensin-converting enzyme (ACE) inhibitors may further block appropriate compensatory autoregulatory responses (Bell 2009) to hypertensive or to orthostatic hypotensive changes in blood pressure and vascular diameter.

Baseline brachial artery diameter (BAD), flow-mediated dilation (FMD), and nitroglycerin-mediated dilation (NMD) have been used as intermediate outcomes in studies of cardiovascular responses to pollution in controlled human exposure (Brook et al. 2002) and observational research (Moens et al. 2005). As predictors of cardiac risk and coronary artery function, BAD (Montalcini et al. 2012) and FMD (Caballero et al. 1999; Gold 2008; Liu et al. 2007; Sullivan et al. 2003) have clinical as well as physiologic relevance. FMD, a measure of the difference in BAD after brachial artery occlusion relative to baseline BAD (before occlusion), reflects the combined effects of endothelial-dependent processes that influence the production and quenching of vasodilatory nitrogen oxide (NO) and endothelial-independent processes that influence vascular smooth muscle responsiveness to NO (Ghio et al. 2000; Salvi et al. 1999; Sørensen et al. 2003). Nitroglycerin-mediated dilation (NMD), a measure of the change in BAD before and after administration of nitroglycerin (an exogenous source of NO), reflects autonomic vascular smooth muscle responsiveness occurring independently of endothelial NO production. A decrease in FMD but not NMD suggests an effect on endothelial function specifically, whereas a decrease in both outcomes suggests that part or all of the change is due to endothelial-independent effects. People with diabetes have markedly reduced but measurable FMD (Caballero et al. 1999). In a previous cross-sectional study of Boston, Massachusetts, USA, residents, we found that short-term increases in traffic and non-traffic pollution were associated with reduced FMD and NMD in participants with type 2 diabetes (O’Neill et al. 2005), suggesting both non-endothelial– and endothelial-mediated mechanisms for pollution-related vascular dysfunction.

High humidity has been associated with hyperpyrexia, a decline in physical strength and fatigue as well as a reduction in alertness and mental capacity. High humidity often occurs when temperature is high, and the effects of the two exposures can be difficult to disentangle (Sharma et al. 1983). In Germany, Schneider et al. (2008b) analyzed the influence of weather parameters [including water vapor pressure (WVP)] on blood pressure, arrhythmia, and ischemia in cardiovascular patients. Schneider et al. (2008b) noted that the associations they detected between weather parameters and ST-segment depression could be relevant to clinical cardiovascular outcomes.

In a prospective, repeated-measures study, we investigated associations of BAD, FMD, and NMD with pollutant exposures, ambient temperature, and humidity in 64 adults with type 2 diabetes.

## Materials and Methods

*Study population and protocol*. The study population consisted of Boston residents with type 2 diabetes mellitus who lived within 25 km of a central air monitoring site located near downtown Boston. Participants were recruited between 2006 and 2009. If they met initial screening criteria, they were invited to come for a baseline visit that included an interview on sociodemographic characteristics, health status, medical history, medication, and lifestyle; blood and urinary analysis; and a clinical examination. This visit provided an opportunity both for further detailed screening and for collecting baseline data on participants who after the visit were found to be eligible for the follow-up study. Exclusion criteria focused on factors that can introduce particle exposure errors (e.g., exposure to second-hand tobacco smoke at home; living > 25 km from the central monitoring station); taking medications with acute vascular effects; having conditions with electrophysiological or vascular effects [i.e., current atrial fibrillation/flutter; history of clinically significant ventricular arrhythmias, a pacemaker or an implanted defibrillator; acute myocardial infarction or stent placement within the last 6 months]; on clinical/biomarker parameters requiring immediate attention [e.g., uncontrolled hypertension (systolic blood pressure > 180 mmHg, diastolic blood pressure > 105 mmHg)]; having markers of poor diabetes/lipid control or advanced diabetic nephropathy [serum cholesterol > 350 mg/dL, serum triglycerides > 600 mg/dL, hemoglobin A1c (HbA1c) > 10.5%; fasting blood glucose > 270 mg/dL; urine albumin/creatinine ratio > 300 μg/dL], and having diagnoses of other advanced diseases (e.g., solid organ transplant, active autoimmune disease, dementia, type 1 diabetes, renal failure, seizure disorder, or stroke).

After entry into the follow-up study, eligible participants completed up to five follow-up clinical examinations scheduled 2 weeks apart on the same weekday in the morning. We ascertained medication use by patient self-report at baseline and at each subsequent visit. The study protocol was approved by the institutional review boards of the Brigham & Women’s Hospital, the Joslin Diabetes Clinic, and the Harvard School of Public Health. All participants provided written informed consent.

At each visit BAD, FMD, and NMD were measured by ultrasound according to standardized protocols. Participants were placed in the supine position with the right arm abducted by approximately 60° and comfortably placed on a support. For the assessment of endothelial-dependent vasodilatation (i.e., FMD), a pneumatic tourniquet was placed on the right forearm 2 cm below the antecubital fossa. After a 15-min rest period, high-resolution brachial artery ultrasound was performed to measure BAD with a 10-MHz linear-array transducer and a Terason 2000 ultrasound (Teratech Corporation, Burlington, MA, USA) with electrocardiogram-gated image acquisition at end diastole. After taking baseline images, the arm was immobilized and the transducer was held in a fixed position throughout the assessment. Reactive hyperemia was produced by inflating the tourniquet to approximately 50 mmHg above the individual’s systolic blood pressure for 5 min and then quickly deflating it (Celermajer et al. 1992). Ultrasound picture acquisition was repeated 60 sec after sudden deflation for 10 sec.

After another 15-min rest period, endothelium-independent vasodilatation (i.e., NMD) was assessed by measuring BAD before and 3 min after the sublingual administration of 0.4 mg nitroglycerin. Blood pressure and heart rate were monitored in the left arm before and after the brachial artery measurements and before and after nitroglycerin administration. NMD was not performed if systolic blood pressure was < 90 mmHg. All acquired ultrasound images were analyzed centrally according to a predefined protocol using Brachial Analyzer Vascular Research Tools 5, version 5.6.12 (Medical Imaging Applications LLC, Coralville, IA, USA). FMD and NMD are expressed as percent change in BAD [i.e., BAD after the intervention (occlusion or nitroglycerin) minus the BAD before the intervention, divided by the BAD before the intervention].

*Environmental data*. Ambient concentrations of fine particle mass [with aerodynamic diameter < 2.5 μm (PM_2.5_)], black carbon (BC), organic carbon (OC), elemental carbon (EC), particle number concentration (PN), and sulfate (SO_4_^2–^) were measured hourly at a central monitoring site (Harvard Supersite) in Boston, Massachusetts. Hourly ambient concentrations of ozone (O_3_), carbon monoxide (CO) and nitrogen dioxide (NO_2_) were estimated by averaging data from the Massachusetts Department of Environmental Protection’s Greater Boston monitoring sites. Missing hourly data for PM_2.5_ and BC were imputed using regression modeling, including a long-term time trend and day of week, hour of day, temperature, relative humidity, barometric pressure, and NO_2_ as predictors. OC, EC, and SO_4_^2–^ were not available in 2006 and for part of 2010; this resulted in approximately 20% missing values. All other exposure variables (both weather parameters and pollution) had few missing values (< 1%), which were assumed to be missing at random.

OC can be both primary and secondary. Using published methods (Lim and Turpin 2002), we estimated primary OC (emitted by cars) by multiplying EC by 1.8, and estimated secondary OC (due to oxidation of traffic emissions as well as biogenic emissions) as OC minus primary OC.

Pollutant exposures were averaged over 24-hr intervals (0900–0900, to correspond to the time of arrival at the study clinic for each visit). In addition, we derived average exposures over the previous 1–6 days when ≥ 75% of daily data were available.

We also estimated individual PM_2.5_ and BC exposures. Five days before each clinic visit, fine particle samplers were placed in each study participant’s home. On the day of the visit, the participant brought the pollution samplers (still in operation) to the clinic. A custom-made Harvard sampling system was used to collect fine particles (i.e., PM_2.5_) on Teflon filters to determine PM_2.5_ and BC mass concentration (by gravimetry and reflectance, respectively) as a measure of 5-day home/trip–integrated exposures. The sampling system also included a SidePak (model AM510; TSI Inc., Shoreview, MN, USA) that provided continuous measurements of PM_2.5_ particle mass concentration, calibrated using the integrated PM_2.5_ filter to derive hourly averages, 24-hr averages, and moving averages of up to 5 days when ≥ 75% of daily (or hourly) data were available.

Weather parameters, including hourly temperature, barometric pressure, and dew point temperature, were obtained from the National Weather Service Station at Logan Airport (Boston, MA, USA), located approximately 12 km from the examination site. WVP (actual water vapor pressure), a measure of humidity defined as the amount of water vapor in a volume of air, increases as the amount of water vapor increases. WVP (hPa) was computed as WVP = 6.1078 × 10^(7.5 × dewpoint)/(237.7 + dewpoint)^ (Barenbrug 1974; Buck 1981; Eaton and Kells 2009). Temperature and WVP were also evaluated as 24-hr averages (0900–0900) and as cumulative averages over the previous 1–5 days.

*Statistical analysis*. We estimated the associations of air pollution and weather with the individual BAD measurements performed during each visit as separate outcomes: BAD before occlusion, BAD after occlusion, BAD before sublingual nitroglycerin administration, and BAD after sublingual nitroglycerin administration. In addition, we estimated associations with FMD (i.e., the percent change in BAD in response to occlusion) and NMD (i.e., the percent change in BAD in response to nitroglycerin administration).

We fit linear models for each outcome that included fixed effects for participant and linear terms for the date of visit, season, and the average temperature on the day of each visit. The indicator variables for each participant control for correlated measurements within subjects, and for confounding by time invariant characteristics such as gender and ethnicity. Baseline and end-of-study glucose control (i.e., HbA1c) was measured using standard methods. When HbA1c was included in our model as a linear variable, it was not a significant predictor of our outcomes, did not improve our model fit, and, therefore, was not included in the final models. Model residuals were normally distributed (data not shown).

Both fixed and random effects approaches to estimation were based on the same model, so the implied correlation structure among repeated measurements is similar in the two models. Hence, a fixed intercept for each participant fully accounts for the within-correlation of the repeated data for each participant. In mixed effects models, the estimated exposure effect is a blend of cross-sectional (across subject) and longitudinal (within subject) comparisons. Because of the cross-sectional component, the estimates can be subject to confounding by characteristics specific to that participant (e.g., socioeconomic status). In contrast, the fixed effect estimates are based purely on longitudinal comparisons, which make them not subject to between-subject confounding. This is the primary reason we used fixed effects in this application (Fitzmaurice et al. 2004). Because we did not want our reported results to be highly dependent on the chosen model, we fit the analogous mixed effects models in sensitivity analyses.

Effect estimates were scaled by the interquartile pollutant range (IQR). Estimates for pollution associations with BAD (before and after occlusion, and before and after sublingual nitroglycerin administration) are presented as a change in BAD in millimeters per IQR increase in pollution. Estimates for pollution associations with FMD and NMD are presented as change FMD or NMD in percent per IQR increase in pollution levels.

We focused on associations with exposures occurring in the 1–6 days before coming to the clinic because of evidence from prior studies (Hoffmann et al. 2012; O’Neill et al. 2005) and biologic priors [vascular autonomic responses to pollutants could be relatively immediate (within 24 hr)], whereas inflammatory vascular responses would be cumulative over a week. With temperature, our previous analytic experience also suggested more immediate responses related to temperature in the 24 hr before the measurement, along with approximately 1-week cumulative responses that could relate effects of persistent episodes of high or low temperature (Hoffmann et al. 2012). We estimated associations of our outcomes with cumulative exposures of up to 14 days, to assess how the associations varied with longer moving averages, and because participants completed clinic visits every 2 weeks. When interpreting our findings, we focused on the consistency of associations among correlated pollutants (e.g., different markers of traffic-related pollution). We estimated the effects of temperature, barometric pressure, and WVP with and without adjustment for particle exposures (5-day average PM_2.5_ and BC), using the same model described above. We first tested the relationship between the meteorological variables and our outcomes with a penalized spline. Because the generalized cross validation (GCV) method estimated one degree of freedom for the penalized spline of the weather parameters and these showed a linear pattern, we then included the meteorological variables linearly in the models.

Results for weather parameters are presented as the change in BAD (in millimeters), FMD and NMD (in percent) associated with an IQR increase in each parameter. To estimate short-term and cumulative effects of weather, we modeled individual daily averages and cumulative averages from the day of the visit up to and including 5 days before the visit.

We examined effect modification by ACE inhibitors and beta-blockers by including an interaction term between the 5-day average of each exposure and the medication variables. Medication use was ascertained by patient self-report at baseline and at each subsequent visit. We also examined effect modification by patient characteristics such as body mass index (BMI) in four categories: underweight (BMI < 18.5); normal (18.5 ≥ BMI < 25); overweight (25 ≥ BMI < 30); obese (BMI ≥ 30), gender, and continuous HbA1c. Finally, we examined effect modification of associations with temperature by season—defined as winter (December–February), spring (March–May), summer (June–August), and autumn (September–November)—by including an interaction term between temperature and season.

In two-pollutant models, we included two pollutants at a time together in each model.

All analyses were performed using R, version 2.14.1 (The R Project for Statistical Computing, Vienna, Austria).

## Results

During the study period (September 2006 to July 2010), 70 participants were enrolled in the repeated-measures study. Of these, 64 had complete covariate information with ≥ 1 acceptable FMD evaluation over a total of 279 visits (Table 1). A subset of 43 participants (159 observations) consented and had blood pressures within a range that allowed us to administer nitroglycerin and measure NMD. The participants had an equal distribution by gender, on average a long-standing history of diabetes (mean, 10 years), and an average BMI of 31 kg/m^2^. The baseline BAD (i.e., the BAD measurement taken before brachial artery occlusion) across all participants ranged between 2.5 and 6.2 mm, with a mean of 4.1 mm (range, 2.5–5.2 mm) in women, and 5.0 mm (range, 3.6–6.2 mm) in men. Although the BAD was comparable to what we have previously measured in healthy adults without diabetes, as expected in our diabetic cohort, the FMD and NMD were markedly reduced (Brook et al. 2009).

**Table 1:** Participant characteristics.

Characteristic	Participants (*n*)	Observations (*n*)	Percent	Mean (range)
Age (years)	64	64		63.9 (45–81)
BMI (kg/m²)	64	64		31.5 (20.5–57.2)
Years with diabetes		62		10.4 (1–38)
Male	32		50
Female	32		50
Medication^*a*^
β-blocker	24		38
Calcium-channel blocker	15		23
ACE inhibitor	29		45
Statin	50		78
Insulin	9		14
Study visits completed
1		64	100
2		60	94
3		57	89
4		54	84
5		44	69
BAD (mm)^*b*^
Baseline	64	279		4.5 (2.5–6.23)
After occlusion	64	279		4.6 (2.6–6.3)
Before nitroglycerin	43	164		4.4 (2.6–6.3)
After nitroglycerin	43	159		4.9 (3.02–6.9)
FMD (%)	64	279		2.0 (–4.6–14.2)
NMD (%)	43	159		10.0 (–0.4–21.3)
Mean values are averaged across all study visits. ^***a***^Medication use was ascertained by patient self-report at baseline and at each subsequent visit. ^***b***^Baseline refers to BAD measured before brachial artery occlusion. BAD before and after nitroglycerine refers to measures taken before and after sublingual administration of nitroglycerine.				

Table 2 summarizes the distribution of the pollutant concentrations and of temperature values for the 24-hr and 5-day averages. For most exposures, data were complete (or nearly so) for all 279 visit days included in the present analysis. However, for OC and EC, 24-hr averages measures were available for only 231 of the 279 days, and for SO_4_^2–^, 24-hr averages were available for 197 days (Table 2). Table S1 in the Supplemental Material shows the correlations among pollutant and meteorological variables, PM_2.5_, BC, OC, EC, and SO_4_^2–^, were highly correlated with each other but were not highly correlated with PN count, O_3_, or temperature. The home/trip–integrated concentrations for BC and PM_2.5_ were not highly correlated with their respective ambient concentrations. The average 24-hr maximum PM_2.5_ concentration (27 μg/m^3^) was below the National Air Quality Standard recommended upper limit of 35 μg/m^3^ (U.S. Environmental Protection Agency 2014).

**Table 2:** Air pollution, temperature, and water vapor pressure among all observations (24-hr average and 5-day average values before each study visit).

Variable	Observations (*n*)	Mean	Percentile			Maximum	IQR
			25th	50th	75th		
Ambient PM_2.5_ (μg/m^3^)
24 hr	278	8.37	5.52	7.38	9.58	26.69	4.06
5 day	279	8.51	6.47	7.61	9.62	21.10	3.14
Indoor continuous PM_2.5_ (μg/m^3^)
24 hr	258	7.11	3.73	5.04	7.96	56.43	4.23
5 day	260	8.93	5.02	7.11	10.51	52.88	5.49
Home/trip–integrated PM_2.5_ (μg/m^3^)
5 day	269	9.18	5.05	7.74	10.70	57.28	5.66
Ambient BC (μg/m^3^)
24 hr	279	0.61	0.41	0.54	0.76	2.62	0.35
5 day	279	0.60	0.48	0.57	0.73	1.25	0.25
Home/trip–integrated BC (μg/m^3^)
5 day	268	0.77	0.56	0.69	0.84	4.89	0.28
OC (μg/m^3^)
24 hr	231	3.03	2.07	2.85	3.82	8.91	1.75
5 day	245	3.03	2.17	2.98	3.78	6.24	1.61
EC (μg/m^3^)
24 hr	231	0.35	0.24	0.30	0.44	0.96	0.20
5 day	245	0.34	0.27	0.34	0.41	0.80	0.14
CO (ppm)
24 hr	279	0.28	0.21	0.27	0.34	1.00	0.13
5 day	279	0.28	0.23	0.28	0.33	0.52	0.10
NO_2_ (ppm)
24 hr	279	0.015	0.011	0.015	0.018	0.033	0.006
5 day	279	0.015	0.012	0.014	0.016	0.025	0.004
O_3_ (ppm)
24 hr	279	0.027	0.020	0.026	0.032	0.061	0.012
5 day	279	0.028	0.022	0.028	0.033	0.047	0.010
PN (1,000/cm^3^)
24 hr	262	13.27	9.03	12.47	17.21	32.67	8.18
5 day	265	12.95	8.92	12.43	16.16	28.39	7.24
SO_4_^2–^ (μg/m^3^)
24 hr	197	2.13	0.95	1.61	2.41	12.34	1.47
5 day	221	2.28	1.37	1.82	2.72	7.08	1.34
Temperature (°C)
24 hr	279	13.71	6.84	14.78	21.20	29.33	14.36
5 day	279	13.68	6.84	15.10	20.99	26.39	14.15
Water vapor pressure (hPa)
24 hr	279	11.41	6.18	10.53	16.32	24.99	10.14
5 day	279	11.49	6.72	10.63	16.06	24.63	9.34
Summary data were based on imputed data for ambient PM_2.5_ and ambient BC.							

**Figure 1:**
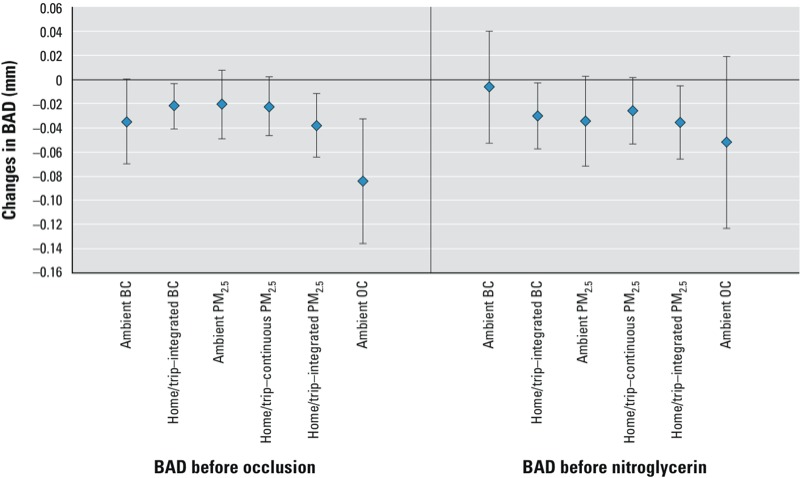
Estimated changes in BAD (in mm) associated with IQR increases in mean 5-day concentrations of each pollutant, including BAD measured at baseline, and before sublingual nitroglycerin administration. Error bars represent 95% CIs.

*Pollution and BAD*. Figure 1 presents the associations of the mean 5-day average concentrations of each of the pollutants with BAD before brachial artery occlusion, and before sublingual nitroglycerin administration. We focus on results related to the 5-day moving averages because we found the largest and most consistent associations of BAD or FMD with the 4 and 5 days moving averages of pollution. A second rationale for this choice is that the indoor/trip–integrated BC and PM_2.5_ were measured from the filters that represented an integrated particle collection over a 5-day period.

Baseline BAD was negatively associated with increases in particle mass and BC and OC particle components (Figure 1). Scaling for the IQR increases in pollution levels, a 0.25 μg/m^3^ increase in 5-day mean ambient BC was associated with a –0.035-mm decrease (95% CI: –0.07, 0.00) in baseline BAD. Baseline BAD also decreased in association with IQR increases in 5-day average exposures to ambient PM_2.5_ (–0.02 mm; 95% CI: –0.05, 0.01, IQR = 3.14 μg/m^3^), ambient OC (–0.08 mm; 95% CI: –0.14, –0.03, IQR = 1.61 μg/m^3^), indoor/trip–integrated BC (–0.02 mm; 95% CI: –0.04, –0.003, IQR = 0.28 μg/m^3^), and indoor/trip–integrated PM_2.5_ (–0.04 mm; 95% CI: –0.06, –0.01, IQR = 5.66 mg/m^3^). Associations with increases in the same exposures during the previous 24 hours, or as cumulative averages over shorter or longer time periods (up to 6 days) (see Supplemental Material, Figure S1) were generally weaker than associations with 5-day average values. To assess for relatively immediate responses on pollution during the trip to the clinic, using the home/trip–continuous PM_2.5_ exposures, we also explored associations of shorter averages of PM (30, 60, and 90 min before the visit) on the day of the visit, but found no associations with our outcomes (data not shown).

Associations of baseline BAD with IQR increases in 5-day mean concentrations of EC, CO, and NO_2_, were negative but nonsignificant, and baseline BAD was not associated with 5-day mean SO_4_^2–^ or O_3_ (see Supplemental Material, Figure S2).

Associations of pollution with brachial artery narrowing tended to weaken after the maneuvers designed to cause brachial artery dilation (shear stress after arterial occlusion or sublingual nitroglycerin). However, despite perturbation of vascular function by the FMD and NMD maneuvers, the previous 5-day exposures to particle pollution tended to have a relatively persistent association with brachial artery narrowing for measures of BAD conducted after occlusion, before or after sublingual nitroglycerin administration (Figure 1; see also Supplemental Material, Figure S3). For example, the previous 5-day home/trip–integrated PM_2.5_ was associated with reduced BAD before and after the FMD maneuver, as well as immediately before the NMD maneuver (see Supplemental Material, Figure S3).

In two-pollutant models, the magnitude and statistical significance of associations of baseline BAD with 5-day mean PM_2.5_ and BC remained essentially unchanged, whereas there were no significant associations with 5-day mean values of NO_2_ or O_3_ based on two-pollutant models (data not shown).

Associations of 5-day mean PM_2.5_ and BC concentrations with baseline BAD were stronger (*p*_interaction_ = 0.01) in participants taking ACE inhibitors. For example, a 3.14-mg/m^3^ increase in 5-day average PM_2.5_ was not associated with baseline BAD among the 35 participants who were not taking ACE inhibitors (0.008 mm; 95% CI: –0.3, 0.04), but baseline BAD was significantly lower among the 29 participants who were taking ACE inhibitors (–0.06 mm; 95% CI: –0.11, –0.02). There was no evidence (*p*_interaction_ = 0.7) of effect modification by beta-blocker use (data not shown).

In sensitivity analyses, we compared fixed with mixed effect models and found that the main results, as well as the effect modification results by medications, were very similar (data not shown). We also found that the associations of BAD and FMD with each pollutant were similar in the smaller group of 42 participants who had both FMD and NMD measures compared with the larger group of 64 participants who had FMD measures (data not shown).

**Figure 2:**
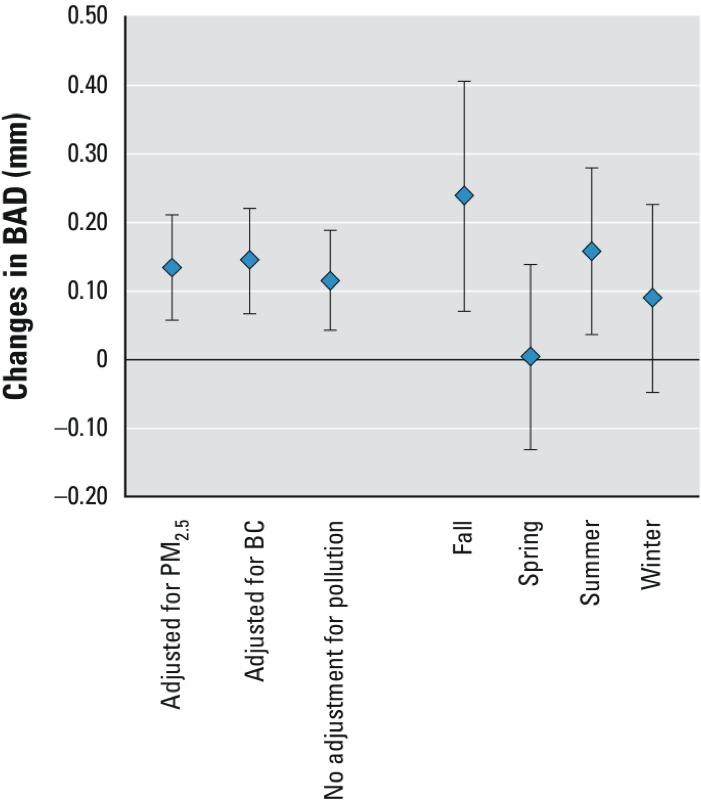
Estimated changes in BAD (in mm) associated with an IQR increase in average daily temperature during the 24 hr before the study visit with and without adjustment for PM_2.5_, or BC by season, for baseline BAD (measured before occlusion). Error bars represent 95% CIs.

*Temperature, humidity, and BAD*. Baseline BAD increased in association with IQR increases in same-day temperature (0.12 mm; 95% CI: 0.04, 0.19, IQR = 14.4°C) and WVP (0.09 mm; 95% CI: 0.02, 0.16, IQR = 10.13 hPa) when estimated using separate models. When temperature and WVP were included in the same model, WVP was no longer significantly associated with baseline BAD (0.06 mm; 95% CI: –0.06, 0.17) and the association with temperature was attenuated (0.09 mm; 95% CI: 0.0, 0.18). However, these estimates are difficult to interpret given the high correlation between temperature and WVP (*r* = 0.88). Associations between temperature and baseline BAD were similar when adjusted for 5-day PM_2.5_ or BC (Figure 2). Associations of temperature with BAD after NMD were also in a positive direction but smaller in magnitude and lower in precision. The associations of same day temperature with baseline BAD were stronger during autumn and summer, and lower in spring and winter (Figure 2).

*Pollution, temperature, humidity, FMD, and NMD*. While the FMD and the NMD tended to increase with increases in pollutant concentrations, the changes with pollution were small, inconsistent, and generally nonsignificant (Table 3). Temperature was not associated with FMD or NMD (data not shown), and barometric pressure was not associated with baseline BAD, FMD, or NMD (results not shown).

**Table 3:** Associations of FMD and NMD with IQR increases (µg/m^3^) in the 5-day mean concentrations of each pollutant.

Exposure	IQR	FMD (95% CI)	NMD (95% CI)
Ambient BC	0.25	0.41 (–0.09, 0.91)	–0.62 (–1.56, 0.31)
Home/trip–integrated BC	0.28	0.38 (0.11, 0.65)	–0.08 (–0.65, 0.49)
Ambient PM_2.5_	3.14	0.37 (–0.04, 0.77)	0.03 (–0.74, 0.80)
Home/trip
Continuous PM_2.5_	5.40	0.08 (–0.25, 0.42)	0.75 (0.20, 1.31)
Integrated PM_2.5_	5.66	0.24 (–0.14, 0.62)	0.62 (0.00, 1.25)
OC	1.61	1.12 (0.43, 1.80)	0.96 (–0.47, 2.38)

## Discussion

As well as demonstrating an association of elevated particle pollution with change in BAD, to the best of our knowledge this is the first longitudinal study of people with type 2 diabetes to estimate associations of BAD with temperature, now recognized in its extremes as a predictor of cardiovascular risk (Montalcini et al. 2012). Our study results suggest that for this cohort of people with diabetes, particle pollution was a brachial artery vasoconstrictor, whereas higher ambient temperature and, perhaps, high humidity were brachial artery vasodilators.

Our study, demonstrating subclinical associations of pollution and weather with changes in BAD, gives us insight into potential mechanisms for the clinical effects of more extreme exposures on populations potentially at risk of particle- and temperature-related health effects. Evidence from multiple epidemiological studies suggests that people with diabetes may be more susceptible to the joint and separate effects of temperature and air pollution on cardiovascular morbidity and mortality (Medina-Ramón et al. 2006; Qian et al. 2010; Schifano et al. 2009; Schwartz 2005; Zanobetti and Schwartz 2001; Zanobetti et al. 2009).

*Potential mechanisms*. BAD is influenced by structural (e.g., atherosclerotic), endothelial-dependent (e.g., NO producing), and endothelial-independent (e.g., autonomic) factors. The short-term associations between environmental exposures and BAD in our study are unlikely to reflect structural effects of the exposures on the vasculature. Our study provides no evidence for endothelial-dependent responses to pollution and weather because there are no consistent FMD responses that are independent of NMD responses. In the absence of supporting evidence of endothelium-specific influences on the brachial artery vasculature, we hypothesize that environmental influences on BAD in the present study are likely endothelial independent, and may, in part, reflect autonomic responses to environmental exposures.

Our data (Figure 1) suggest that the dominant effects of pollution on reduction of BAD are relatively constant even after the FMD maneuver, particularly 15 min later when, before nitroglycerin administration, the brachial artery has had time to recover from the shear stress maneuver and come back towards its “baseline” diameter for the day of observation. Because the dominant and persistent influence of particle pollution appears to be to narrow the brachial artery, we do not interpret the small and inconsistent pollution-associated increases in FMD or NMD to be “beneficial.” We interpret the small, inconsistent and transient particle pollution-associated increases in FMD or NMD that we and others have seen (Liu et al. 2007; Peretz et al. 2008) to be mathematically a function of the transient increase in arterial diameter occurring after the sheer stress or NMD maneuvers, in the context of a relatively constant influences of pollution on BAD narrowing.

Mean FMD in this diabetic population (2%) was lower than what we have measured in healthy adult populations [e.g., approximately 3.6% by Brook et al. (2009)]; this functional impairment is well documented and makes discernment of specific influences on FMD/endothelial function in this population challenging, with a higher coefficient of variation of FMD compared with BAD (Montalcini et al. 2012). Nevertheless, in similar populations of people with diabetes in Boston, in a placebo-controlled randomized trial of 87 people, we were able to measure changes in FMD in response to troglitazone, an insulin-sensitizing agent (Caballero et al. 2003). In a smaller trial of 24 people, exercise and weight reduction were associated with increased FMD (Economides et al. 2004, 2005; Hamdy et al. 2003).

Whereas most controlled human exposure chamber studies have shown concentrated ambient fine particle associations with reduction in BAD, chamber- (Brook et al. 2002, 2009; Peretz et al. 2008; Urch et al. 2005) and community-based (Briet et al. 2007; Dales et al. 2007; Schneider et al. 2008a) studies evaluating pollution effects on FMD in diabetic and nondiabetic populations have reported inconsistent findings. Differences in study design, population sensitivity, particle composition-related toxicity (Brook et al. 2009), and the higher coefficient of variation in FMD compared with BAD (Montalcini et al. 2012) have been cited as possible explanations for differing study results. In a previous Boston study (O’Neill et al. 2005), we found cross-sectional (between-person) associations of PM_2.5_, BC, and SO_4_^2–^ with reduced FMD and NMD in people with diabetes but not in people without diabetes. These results supported an endothelium-independent component to the vascular responses to pollution from traffic (e.g., BC) as well as non-traffic (e.g., SO_4_^2–^) sources in people with diabetes. In a study in Paris, France, of healthy males breathing ambient air, Briet et al. (2007) also found that pollution from non-traffic (i.e, SO_4_^2–^) and traffic (i.e, NO) was associated with reduced FMD. However, in contrast to the study by O’Neill (2005), NMD was not associated with either pollutant, supporting the possibility of endothelium-mediated mechanisms for pollution effects in this small nondiabetic cohort (*n* = 40). This possibility was also supported by a prospective repeated-measures study in North Carolina (Schneider et al. 2008a) in which people with diabetes (*n* = 22) had decreased FMD in association with increased PM_2.5_, but PM_2.5_ was not significantly associated with NDM. Likely because of concern of potential adverse effects of NMD, many controlled human exposure and community-based studies have not measured NMD as a control (e.g., Dales et al. 2007), making it uncertain whether an FMD response is endothelial dependent or not.

*Sources of pollution and BAD narrowing*. As in some of the European and North American studies mentioned above, our data suggest that pollution from non-traffic as well as traffic sources may influence vascular outcomes. PM_2.5_, BC, and OC had the strongest negative associations with BAD, whereas other exposures such as particle number concentration and gaseous pollutant concentrations showed no or less consistent associations with arterial diameter. The bulk of the OC associations that we found came from secondary OC [effect estimate: –0.07 (95% CI: –0.11, –0.02)], whose sources are both oxidation of traffic emissions and other biogenic emissions (Lim and Turpin 2002), rather than primary OC [effect estimate: –0.01 (95% CI: –0.05, 0.03)], whose primary source is motor vehicles.

Previously, we have demonstrated associations of pollution and temperature with changes in blood pressure (Hoffmann et al. 2012). However, despite the associations of pollution and temperature with both BAD and blood pressure, these two outcomes were only weakly correlated (*r* = 0.08 and *r* = 0.07 for correlations of BAD and systolic or diastolic blood pressure, respectively). This is not surprising because BAD is measured in a medium-sized conduit artery, whereas blood pressure is mainly determined by cardiac output times the resistance of the arterioles, which were not observed directly. Thus, the constriction and dilation of these two sections of the vascular system may be subject to both shared and disparate physiologic mechanisms.

People with diabetes have impaired regulation of vascular tone in both the micro- and macrovasculature. Homeostatic vascular responses to changes in ambient temperature and other environmental stimuli may be impaired in people with diabetes because of impaired autonomic regulation, fluid shifts, and medication effects. We did not find significant departures from linearity for the relationship of temperature and BAD, suggesting that medium conduit artery vasoconstriction occurs at lower temperatures as well as vasodilation at high temperatures. In diabetic patients, either extreme may be a cardiac risk factor consistent with the U-shaped relation of temperature with cardiac mortality in very cold as well as very hot weather (Wolf et al. 2009).

Our study was limited in size, limiting the power to evaluate effect modification by personal characteristics of this diabetic cohort. Our study also had limitations related to the precision of outcome and exposure measurement. Although estimation of “peak diameter” by evaluating post-occlusion diameter at a single point in time—60 sec after the cuff release—is a valid approach dating back to the classic work of Celermajer et al. (1992), recent work published after that study suggests that it may result in an underestimation of FMD, with potential for type II error (Thijssen et al. 2011). Some recent studies have chosen to define the peak diameter after cuff release, allowing the time to peak diameter measure to vary (Thijssen et al. 2011). Some of our study participants worked, and were not home during the day, so that the measurements are not called “personal,” but rather home/trip measurements, and have their limitations as estimates of personal exposure. Nevertheless, this study design resulted in personal exposure measurements during travel to the clinic, immediately before outcome measurements.

## Conclusions

Air pollution is a leading cause of mortality and morbidity (Lim et al. 2012). Observational studies have shown that rates of cardiovascular hospitalizations and death in association with particle pollution (Zanobetti and Schwartz 2001; Zanobetti et al. 2009) and extreme ambient temperatures (Medina-Ramón et al. 2006; Schifano et al. 2009; Schwartz 2005) are increased in people with diabetes compared with the general population.

Our findings suggest that in people with type 2 diabetes, particle pollution may cause vasoconstriction of medium-sized conduit arteries, whereas higher ambient temperature and WVP may cause vasodilation. Our study provides insight into potential mechanisms for the clinical associations of more extreme environmental exposures on people with type 2 diabetes, a disease resulting in reduced ability to effectively respond to environmental perturbation of vascular responses relevant to cardiac risk.

## Supplemental Material

(156 KB) PDF
