# Supplementary material for: Brachial Artery Responses to Ambient Pollution, Temperature, and Humidity in People with Type 2 Diabetes: A Repeated-Measures Study
Source: Environ Health Perspect. 2014 Jan 7;122(3):242–8. doi: 10.1289/ehp.1206136 (PMC3948021; doi:10.1289/ehp.1206136)
Supplement: (156 KB) PDF [file ehp.1206136.s001.pdf]

Environmental Health Perspectives doi:10.1289/ehp.1206136

Supplemental Material
Brachial Artery Responses to Ambient Pollution, Temperature, and Humidity in People with Type 2 Diabetes: A Repeated-Measures Study

Antonella Zanobetti, Heike Luttmann-Gibson, Edward S. Horton, Allison Cohen, Brent A. Coull, Barbara Hoffmann, Joel D. Schwartz, Murray A. Mittleman, Yongsheng Li, Peter H. Stone, Celine de Souza, Brooke Lamparello, Petros Koutrakis, and Diane R. Gold

Table of Contents

Supplemental Material, Table S1 page 2

Supplemental Material, Figure S1 page 3

Supplemental Material, Figure S2 page 4

Supplemental Material, Figure S3 page 5

Supplemental Material, Table S1. Correlations among the pollutants and meteorological variables.

	**Ambient $PM_{2.5}$ (μg/m^3)**	**Indoor continuous $PM_{2.5}$ (μg/m^3)**	**Home/trip integrated $PM_{2.5}$ (μg/m^3)**	**Ambient BC (μg/m^3)**	**Home/trip integrated BC (μg/m^3)**	**OC (μg/m^3)**	**EC (μg/m^3)**	**PN (1000/cm^3)**	**SO_4 (μg/m^3)**	**CO (ppm)**	**NO_2 (ppm)**	**O_3 (ppm)**	**Temperature (°C)**	**Water vapor pressure (hPa)**
Ambient $PM_{2.5}$ (μg/m^3)	1	0.39	0.12	0.65	0.08	0.54	0.55	-0.16	0.76	0.34	0.35	0.36	0.32	0.42
Indoor continuous $PM_{2.5}$ (μg/m^3)		1	0.44	0.42	0.14	0.24	0.32	0.05	0.47	0.34	0.27	0.00	0.11	0.19
Home/trip integrated $PM_{2.5}$ (μg/m^3)			1	0.18	0.49	0.07	0.13	0.11	0.13	0.10	0.18	-0.17	0.05	0.06
Ambient BC (μg/m^3)				1	0.20	0.50	0.86	0.05	0.52	0.52	0.53	-0.07	0.27	0.37
Home/trip integrated BC (μg/m_3)					1	0.06	0.07	0.16	0.07	0.07	0.21	-0.19	-0.07	-0.09
OC (μg/m^3)						1	0.55	-0.15	0.43	0.32	0.30	0.26	0.25	0.22
EC (μg/m^3)							1	0.06	0.53	0.45	0.53	-0.05	0.26	0.35
PN (1000/cm^3)								1	-0.27	0.25	0.56	-0.39	-0.76	-0.72
SO_4 (μg/m^3)									1	0.22	0.20	0.33	0.33	0.45
CO (ppm)										1	0.58	-0.05	-0.10	0.00
NO_2 (ppm)											1	-0.26	-0.34	-0.33
O_3 (ppm)												1	0.35	0.27
Temperature (°C)													1	0.88
Water vapor pressure (hPa)														1

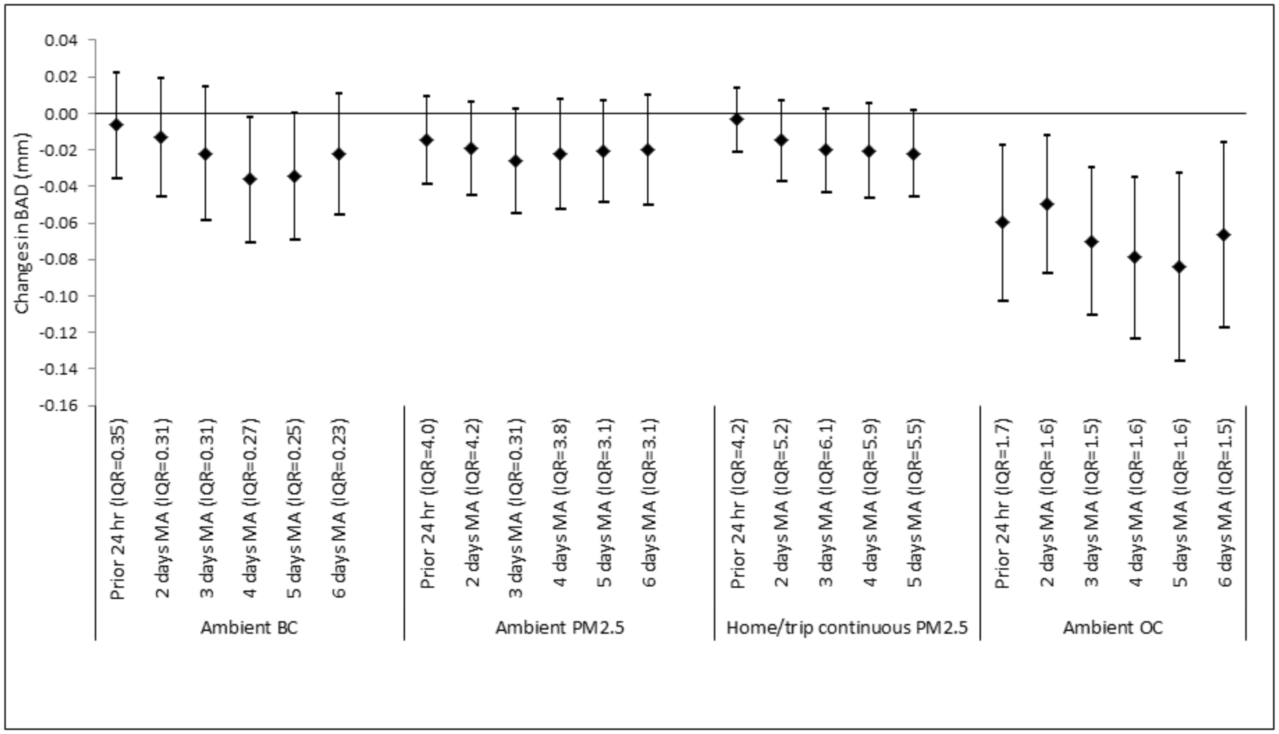

Supplemental Material, Figure S1. Change in mm (95% CI) in brachial artery diameter (BAD) before occlusion for an IQR increase in the prior 24 hours and for moving averages (MA) over the 2- to 6-days before the study visit of each pollutant.

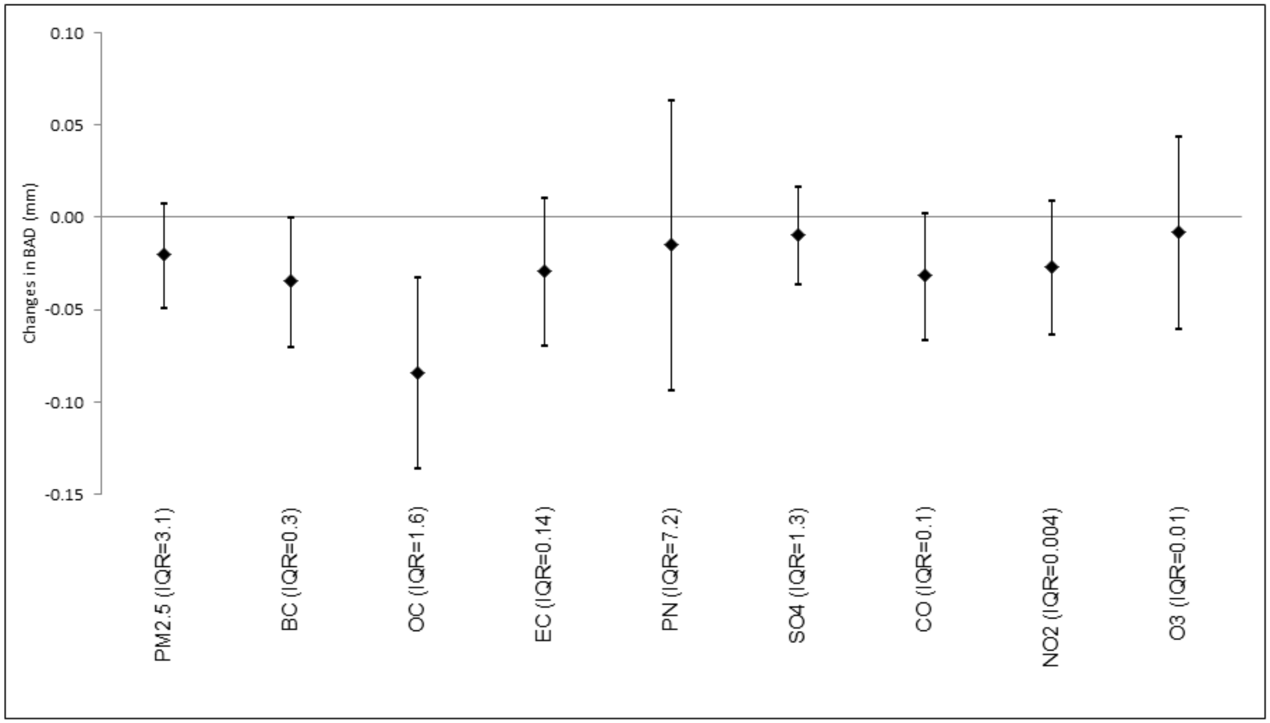

Supplemental Material, Figure S2. Change in mm (95% CI) in brachial artery diameter (BAD) before occlusion for IQR increases in 5-day mean concentrations of each pollutant prior to the study visit.

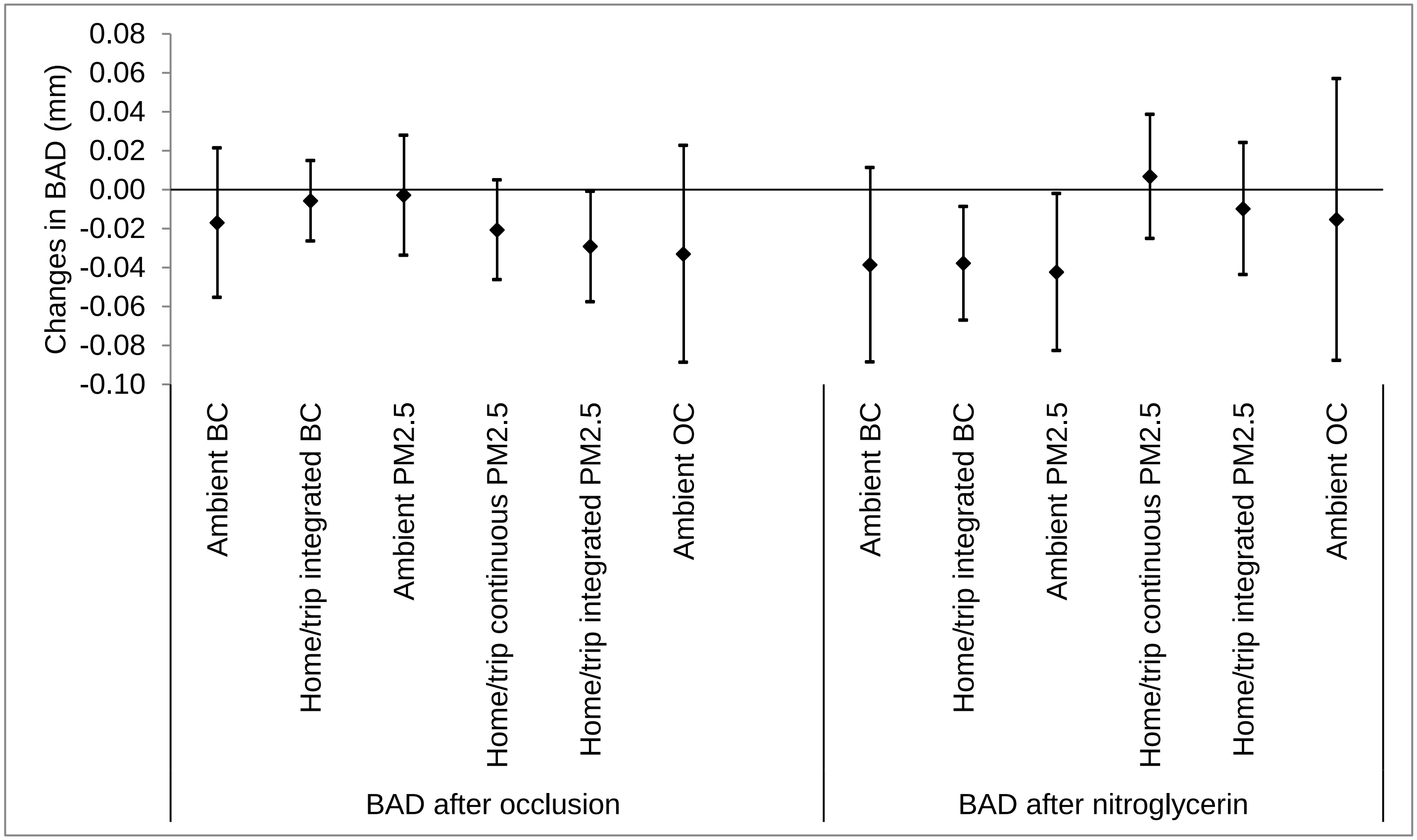

Supplemental Material, Figure S3. Change in mm (95% CI) in brachial artery diameter (BAD) after occlusion and after sublingual nitroglycerin for an IQR increase in 5-day mean concentrations of each pollutant prior to the study visit.
